# Study of Photodegradation of Bentazon Herbicide by Using ZnO-Sm_2_O_3_ Nanocomposite Under UV Light

**DOI:** 10.3390/ijms252413319

**Published:** 2024-12-12

**Authors:** Sadaf Yasmeen, Luca Burratti, Leonardo Duranti, Antonio Agresti, Paolo Prosposito

**Affiliations:** 1 Industrial Engineering Department, University of Rome Tor Vergata, Via del Politecnico 1, 00133 Rome, Italy; sadaf.yasmeen@students.uniroma2.eu (S.Y.); paolo.prosposito@uniroma2.it (P.P.); 2 Department of Engineering and Sciences, Mercatorum University, Piazza Mattei 10, 00186 Rome, Italy; 3 Department of Chemical Science and Technologies, University of Rome Tor Vergata, Via Della Ricerca Scientifica 1, 00133 Rome, Italy; leonardo.duranti@uniroma2.it; 4 Department of Electronic Engineering, University of Rome Tor Vergata, Via del Politecnico 1, 00133 Rome, Italy; antonio.agresti@uniroma2.it

**Keywords:** ZnO-Sm_2_O_3_ nanocomposite, nanoparticles, bentazon, photocatalytic activity, advance oxidation process, wastewater treatment

## Abstract

The removal of organic pollutants from water is significantly important as they have harmful effects on the ecosystem. Heterogeneous photocatalysis is a potential technique for the removal of organic pollutants from the wastewater. In this article, zinc oxide (ZnO) and samarium oxide (Sm_2_O_3_) nanoparticles and ZnO-Sm_2_O_3_ nanocomposite (ZS) were synthesized by the co-precipitation method. We report the bandgap engineering of zinc oxide (ZnO) by making a composite with samarium oxide (Sm_2_O_3_) to enhance the photocatalytic activity. The smaller optical energy bandgap of the ZS nanocomposite as compared to the individual oxide nanoparticles shows that it has a light absorption range from UV to natural light. The photodegradation of bentazon herbicide as a model pollutant has been investigated by using the prepared samples. The photocatalytic activity of the prepared sample against bentazon herbicide was carried out under UV light for 140 min. The degradation efficiency against bentazon of the prepared samples was ZS > ZnO > Sm_2_O_3_, respectively. The ZnO-Sm_2_O_3_ nanocomposite showed a higher photocatalytic performance against bentazon and achieved a 90% degradation efficiency under a UV light source in 140 min. The pseudo-first-order degradation kinetic was studied under different operational conditions, such as catalyst loading, initial pH and bentazon concentration, showing that the degradation rate of bentazon was strongly influenced by these operational parameters. The obtained optimization conditions for practical application were a catalyst loading of 20 mg, pH of solution equal to 7 and bentazon concentration of 5 ppm for ZS nanocomposite in 60 mL of contaminated water. Furthermore, based on the scavenger study, hydroxyl and superoxide radicals play major role in the degradation experiment. The obtained results show that ZS nanocomposite can be a good potential candidate for wastewater treatment.

## 1. Introduction

Over the last few decades, water pollution has become a worldwide issue due to modern agriculture and the occurrence of micropollutants in aquatic environments, which has harmful effects on living beings. These micropollutants may include industrial chemicals, pharmaceuticals, pesticides, herbicides, personal care products and many other emerging compounds. The release of these micropollutants without any pretreatment is one of the main reasons for aquatic pollution. In Europe, the main forms of contamination in drinking water supplies are herbicides and pesticides. A variety of herbicides are significantly used in modern agriculture for the better growth and control of weeds [1,2]. Many herbicides are highly toxic and dangerous even at low contents and are not degraded into nontoxic products easily, representing challenges for wastewater treatment process. Nowadays, bentazon is one of the selective herbicides that is used as a post-emergence herbicide to control weeds in rice, soyabean, cereals, etc. Abernathy and Wax [1] reported that bentazon at natural pH exhibits high mobility in soil. It has high water solubility, due to which it is not retained by soil and moves easily in underground water, contributing to pollution. In this regard, the US Environmental Protection Agency (USEPA) and World Health Organization (WHO) established the threshold levels of herbicides in drinking water at approximately 30 μg/L [3].

Various sustainable technologies and green methods have been employed for the effective removal of organic and inorganic compounds such as herbicides and heavy metal ions from polluted water [4,5]. One of the effective and economical techniques for water purification is heterogeneous photocatalysis, which is used to degrade toxic agrochemical substances including herbicides and pesticides in the presence of natural sunlight and artificial light [6,7]. Metal oxide-based semiconductor photocatalysts have gained much attention for the photocatalytic degradation of toxic substances from wastewater because of their small bandgap energy, ease of synthesis and improved physicochemical properties. Therefore, the fabrication of new nanomaterials for wastewater treatment using transition metals and rare earth oxides is an interesting and popular research field [8,9,10,11].

Among numerous available transition metals and rare earth oxide semiconductors, zinc oxide (ZnO) has a bandgap energy of 3.37 eV, and it is an efficient photocatalyst due to its unique optical, chemical and electrical properties [12]. Additionally, Sm_2_O_3_ having a wide bandgap energy of 4.5 eV and good optoelectronic properties is attractive for the fabrication of solar cells, heterogenous photocatalysis, biosensors and fuel cells [13,14]. In photocatalytic applications, the main hindrance of using individual oxides is their large energy bandgap, which restricts the creation of reactive oxygen species (ROS) and decreases the photocatalytic degradation efficiency. Several techniques have been adopted to improve photocatalytic and physical properties of these materials, like bandgap tuning, doping, co-doping and combining different bandgap oxides to make nanocomposites. The fabrication of nanocomposites by coupling different energy bandgap transition metal oxides and rare earth metals is an effective method to enhance the properties of the final photocatalyst [15,16]. The coupling of large energy bandgap materials, such as ZnO and Sm_2_O_3_, hinders the photoinduced electron-hole recombination and increases the charge carriers transportation, which enhances the photocatalytic characteristics [13]. Many researchers fabricated binary and ternary nanocomposites of rare earth and transition metal oxides for improved photocatalytic properties of individual oxides such as [ZnO-Y_2_O_3_], [ZnO-Er_2_O_3_], [Sm_2_O_3_-ZnO], [ZnO-Sm_2_O_3_-Y_2_O_3_], [ZnO-Ho_2_O_3_-Sm_2_O_3_], etc. [17,18,19,20,21].

However, to the best of our knowledge, the photocatalytic degradation of bentazon has not been reported by using a binary nanocomposite of transition metal oxides and rare earth metals or even individual rare earth metals. In the present study, we reported on the synthesis of ZnO, Sm_2_O_3_ nanoparticles (NPs) and ZnO-Sm_2_O_3_ (ZS) nanocomposite via co-precipitation technique. Structural properties were investigated by using X-ray diffraction, while optical properties were studied by through UV-Visible and photoluminescence spectroscopies. The photocatalytic activity of synthesized samples was evaluated against the bentazon herbicide under UV light. The pseudo-first-order degradation kinetic was studied under different operational conditions, such as the type of photocatalyst, catalyst loading, initial pH and bentazon concentration, to determine the best conditions for bentazon degradation. Moreover, the role of active species in photocatalytic experiments was evaluated by scavenger study.

## 2. Results and Discussion

### 2.1. X-Ray Diffraction

**Figure 1:**
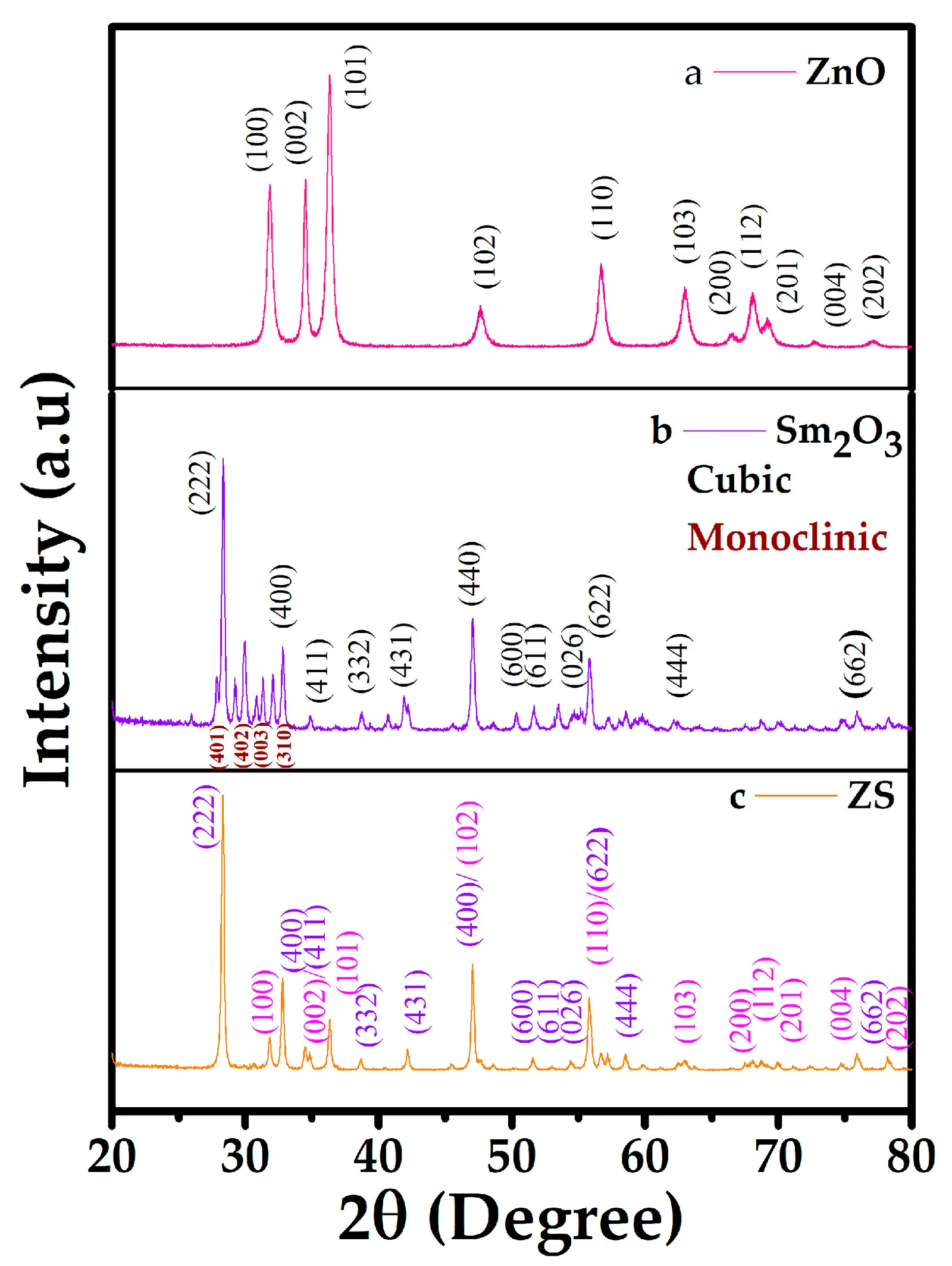
XRD spectra of (**a**) ZnO, (**b**) Sm_2_O_3_ nanoparticles and (**c**) ZnO-Sm_2_O_3_ nanocomposite.

The crystal structure and phase purity of ZnO, Sm_2_O_3_ NPs and ZS nanocomposite was investigated through XRD analysis in the range of 2θ = 20–80°, as shown in Figure 1. ZnO NPs show a hexagonal structure (JCPDS card #36–1451), while the diffraction peaks of Sm_2_O_3_ NPs marked in black color are compatible with a cubic phase structure (JCPDS card #86–2479) and the peaks marked in wine color are attributed to a monoclinic phase structure (JCPDS card #76–0601) of samarium oxide, which is consistent with the literature. In ZS nanocomposite, all the characteristic peaks are compatible with the hexagonal phase of ZnO and cubic phase of Sm_2_O_3_, which indicates that the presence of ZnO in the synthesis process inhibits the formation of the monoclinic phase of Sm_2_O [19]. Furthermore, the cubic structure of samarium has better physicochemical properties as compared to monoclinic structure [14]. The presence of two phases in ZS nanocomposite confirmed the successful synthesis of binary metal oxide ZnO-Sm_2_O_3_ nanocomposite. In XRD patterns, there is no extra peak, indicating the high phase purity of all the prepared samples. The average crystallite size of prepared samples was calculated from the Debye Sheerer formula as follows [22]:

(1) $$D=\frac{K\lambda }{\beta_{hkl}\;\cos\;Ɵ}$$

The calculated crystallite size and microstructural parameters such as lattice constants (*a*, *c*), unit cell volume (*v*), d-spacing (*d*) and dislocation density (*δ*) [23] were calculated and are listed in Table 1. The small variation in the structural parameters can be ascribed to the mutual interactions of Zn^2+^ ions and Sm^3+^ ions in ZS nanocomposite. The crystallite size and dislocation density are inversely proportional to each other. The calculated crystallite size and dislocation density influence the photocatalytic activity. As the dislocation density increases, it hinders the grain growth and increases the photocatalytic activity by inhibiting the recombination rate of charge carriers [24,25]. ZnO has a higher photocatalytic activity as compared to Sm_2_O_3_, due to a smaller crystallite size and higher dislocation density. In ZS nanocomposite, the ZnO and Sm_2_O_3_ follow the same trend. Also, in nanocomposite, the photocatalytic activity is higher due to many factors such as the lower bandgap, synergistic effect between Zn and Sm, lower recombination rate and higher charge carrier transportation [26].

### 2.2. Optical Analysis

**Figure 2:**
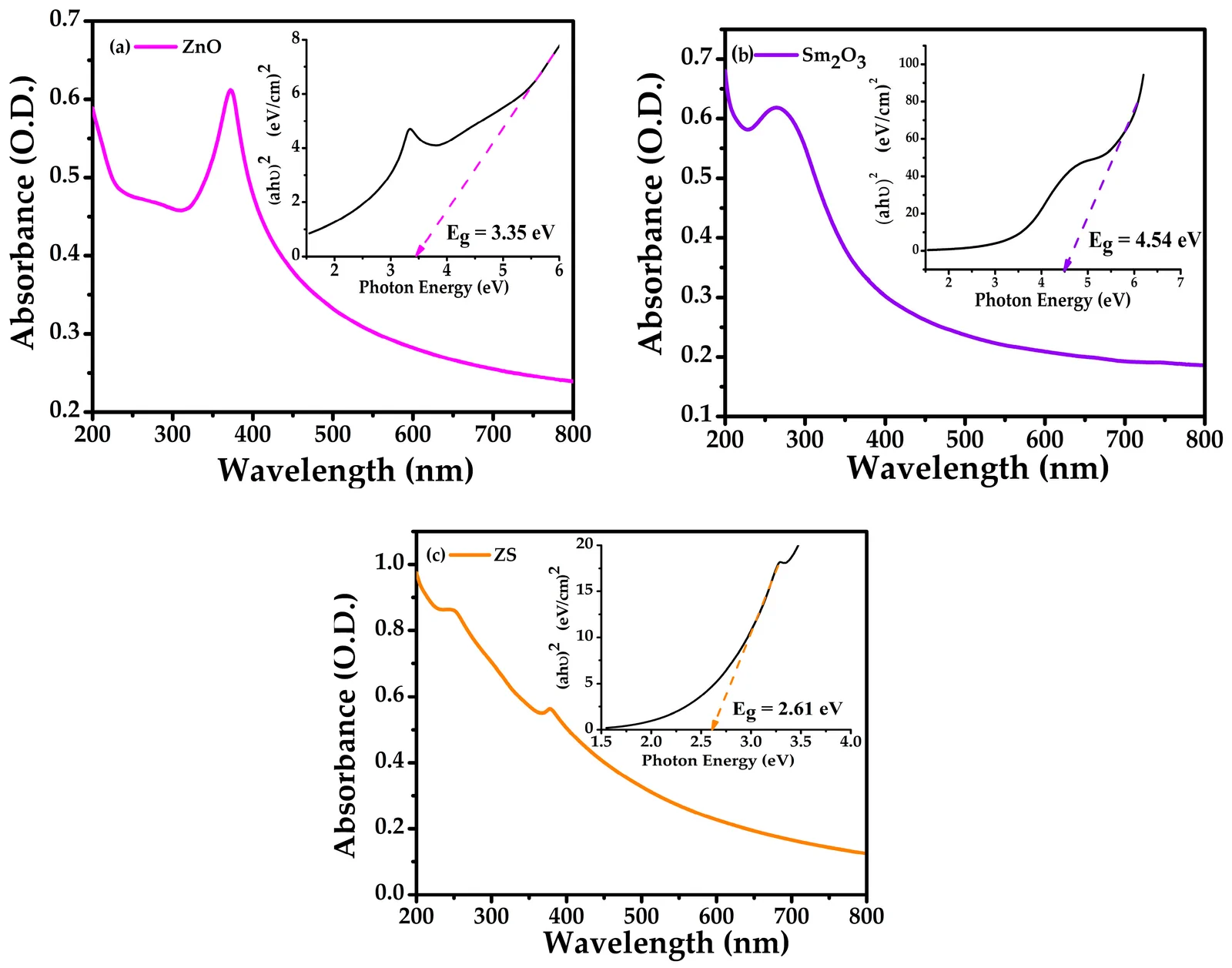
UV-Vis absorption spectra of (**a**) ZnO (reprinted from Figure 5b in previous article [7]), (**b**) Sm_2_O_3_ nanoparticles and (**c**) ZS nanocomposite; insets show Tauc’s plot of prepared samples for energy bandgap estimation.

Figure 2a–c displays the UV-Vis absorption spectra of ZnO, Sm_2_O_3_ NPs and ZS nanocomposite in the range of 200–800 nm. For ZnO, Sm_2_O_3_ nanoparticles maximum absorption was observed at a wavelength of 371 nm and 261 nm, respectively. In ZS nanocomposite, the absorption edges at 380 nm (ZnO) and 250 nm (Sm_2_O_3_) are due to the individual oxide phases, confirming the two oxides in a single matrix [27] as shown in Figure 2c. The optical bandgap of all prepared samples was calculated by the Tauc plot method by using the following equation [28]:

(2) $$\alpha h\upsilon =A{\left(h\upsilon -E_{g}\right)}^{1/2}$$

where *h* is the Planck’s constant, υ is the incident light frequency, *A* is a constant and *E_g_* represents the bandgap energy, respectively.

The bandgap energy for the prepared samples was calculated by drawing a graph between (αhυ)^2^ and photon energy (hυ) and extrapolated from the linear portion to (αhυ)^2^ = 0 as shown in Figure 2 (insets). The calculated bandgap values of ZnO, Sm_2_O_3_ NPs and ZS nanocomposite were 3.35 eV, 4.54 eV and 2.61 eV, respectively [20,29,30]. The calculated bandgap of the ZS nanocomposite was lower as compared to the individual oxide nanoparticles. The decrease in the bandgap in nanocomposite might be due to the average crystalline size and quantum confinement effect or the mutual interaction of ions such as the substitution of Sm^2+^ into ZnO lattice and vice versa as consequence of the different ionic radii of Zn^2+^ (0.74 Å) and Sm_2_O_3_ (0.96 Å). These substitutions create a heterojunction that improves the charge carrier separation [31,32]. Furthermore, due to the difference in the valence and conduction band between ZnO and Sm_2_O_3_, the coupling of these oxides promotes charge carrier generation and inhibits the electron-hole pair recombination, which leads to enhancing the photocatalytic activity [33]. Hence, the small bandgap of nanocomposite makes it a suitable candidate for photocatalytic applications.

**Figure 3:**
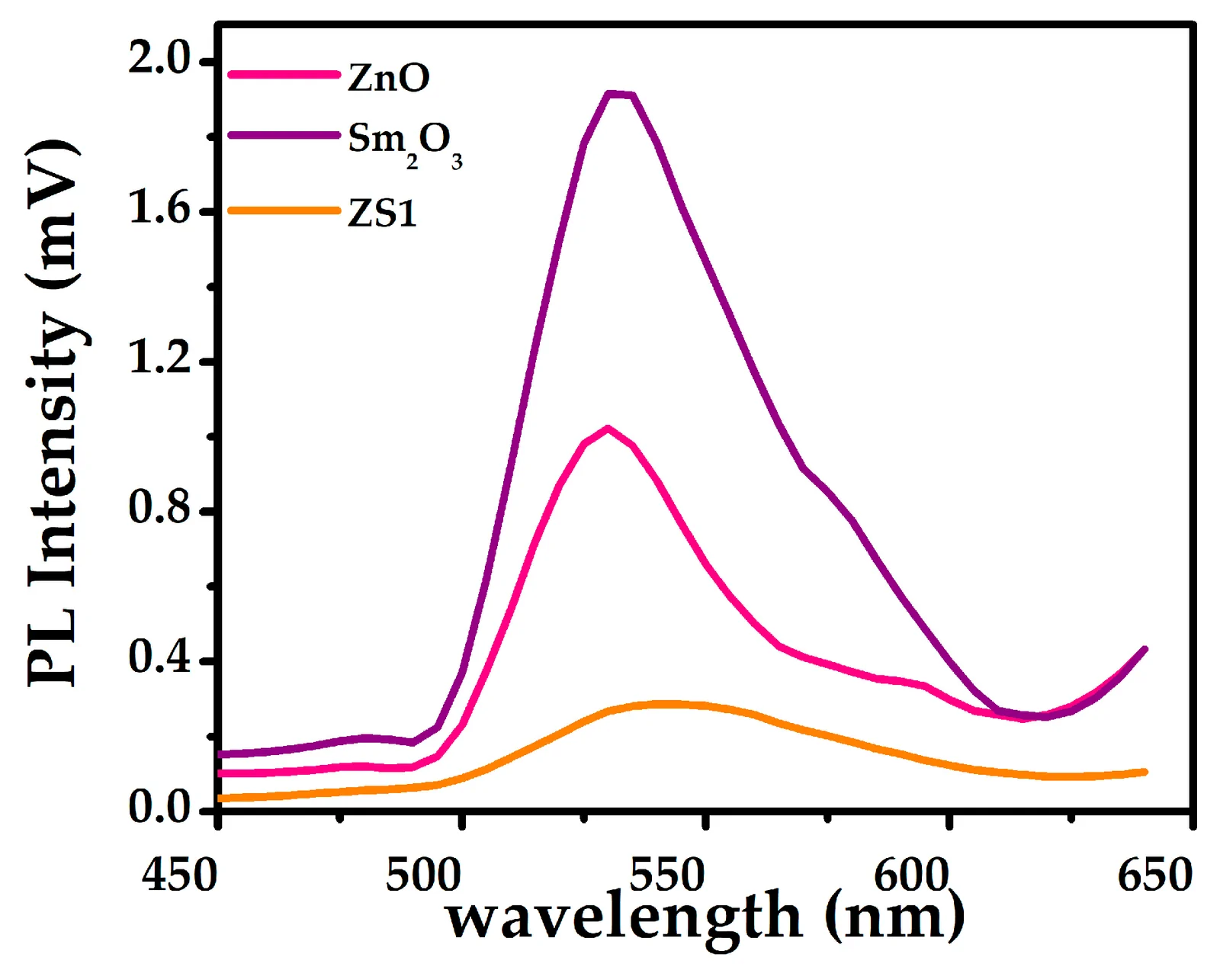
PL spectra of ZnO, Sm_2_O_3_ nanoparticles and ZS nanocomposite.

The PL spectroscopy of ZnO, Sm_2_O_3_ NPs and ZS nanocomposite was carried out at room temperature at an excitation wavelength of 340 nm in the range of 450–640 nm as shown in Figure 3. The PL spectrum showed emission peaks at 528 nm, 532 nm and 537 nm for ZnO, Sm_2_O_3_ and ZS nanocomposite, respectively. In ZnO and Sm_2_O_3_ NPs, the emission bands can be ascribed to oxygen vacancy defects or may be due to native defects such as zinc and samarium interstitials. In photocatalytic reactions, oxygen vacancies and defects can become centers to capture photogenerated electrons, which inhibit the recombination of photoinduced electrons and holes. Moreover, the oxygen vacancies favor the adsorbed O_2_ to trap the photo-induced electron, instantaneously producing $O_{2}^{•-}$ radical groups [34,35]. These $O_{2}^{•-}$ radicals react with pollutants and make byproducts, as discussed in Section 2.4.5. In ZS nanocomposite, the emission peaks are due to oxygen vacancies and defects. However, due to the formation of the heterojunction in nanocomposite, there is an increase in th eelectron transfer rate between ZnO and Sm_2_O_3_, which inhibits the recombination and improves the charge carriers separation and enhances the photocatalytic performance [36,37,38]. The broad and varied intensity of peaks in the samples is due to the combination of several recombination sites and defects in the vacancy level of oxygen and zinc [39].

### 2.3. SEM Analysis

**Figure 4:**
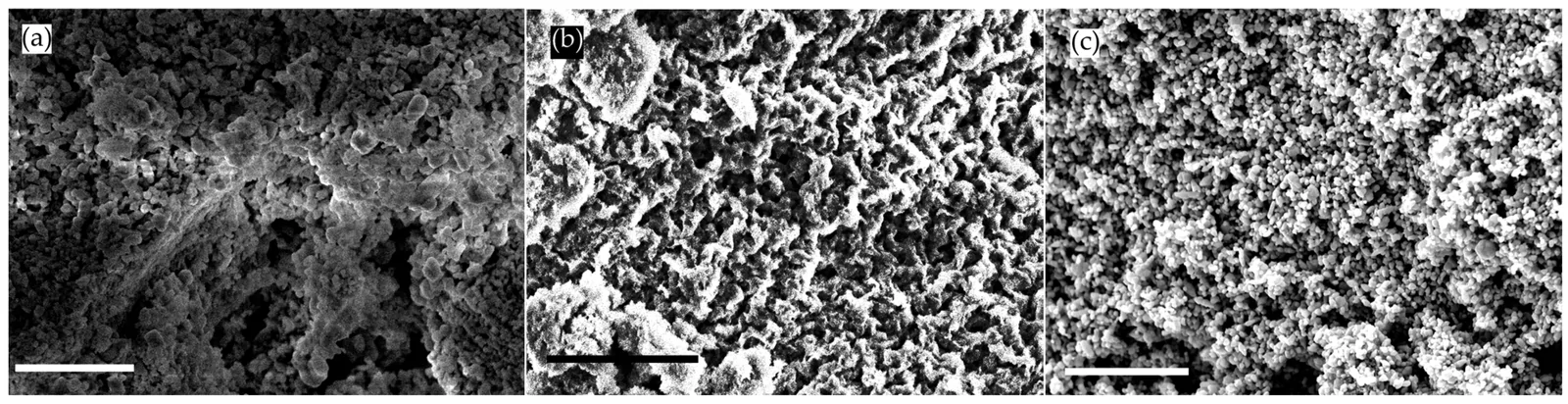

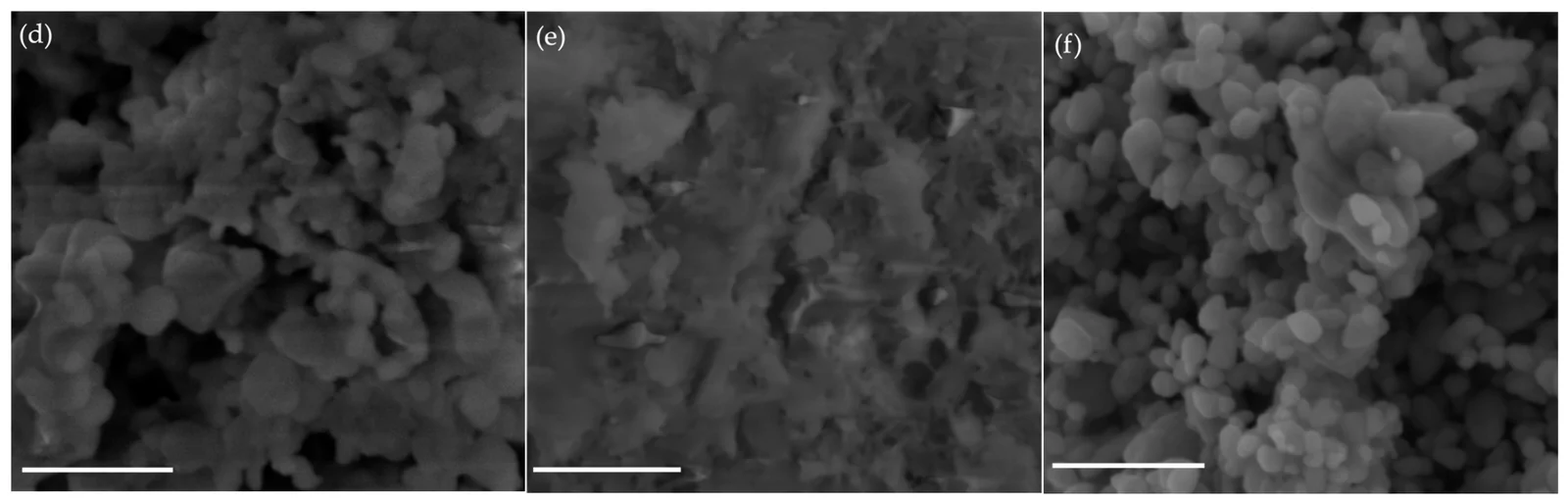
SEM images of ZnO (**a**,**d**), Sm_2_O_3_ (**b**,**e**) and ZS nanocomposite (**c**,**f**); scale bar for (**a**–**c**) is 2 µm and for (**d**–**f**) is 500 nm.

Figure 4 shows the SEM images of the three samples, ZnO, Sm_2_O_3_ and composite, at different magnifications. The sample ZnO presents an irregular structure (Figure 4a); this is also observed in (Figure 4d), where the particles have a jagged shape. The Sm_2_O_3_ sample, on the other hand, shows a flake structure of large dimensions (Figure 4b), and by increasing the magnification (Figure 4e), the structure consists of a compact layer of material. The composite (ZS) appears very different from the first two; in fact, this mainly consists of particles that are much more regular in size and shape, as can be observed from (Figure 4c,f). The reduced particles size of the composite has a larger surface area; consequently, the active area for photocatalysis is larger than that of other samples, playing a key role in the degradation of the herbicide.

### 2.4. Photocatalytic Activity

**Figure 5:**
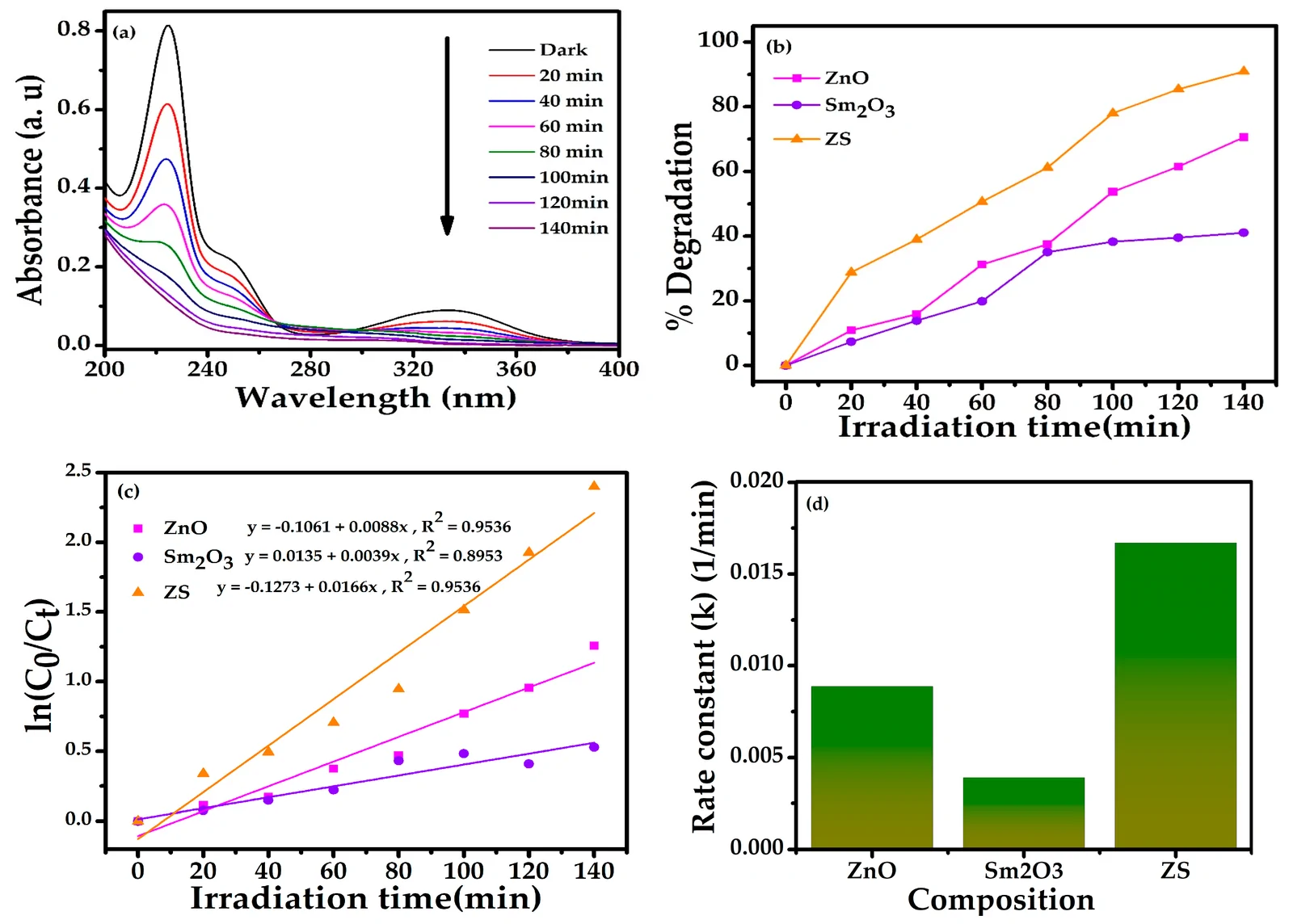
(**a**) Absorption spectra of bentazon in the presence of ZS nanocomposite; (**b**) degradation efficiency at different time intervals against bentazon for Zs, ZnO and Sm_2_O_3_; (**c**) degradation rate; (**d**) rate constant values (*k*) in the presence of ZnO, Sm_2_O_3_ nanoparticles and ZS nanocomposites.

The photocatalytic activity of ZnO, Sm_2_O_3_ NPs and ZS nanocomposite was investigated by using bentazon herbicide as a model organic pollutant under UV light. The change in the concentration of bentazon in water was recorded from absorption spectra after continuous time interval of 20 min. The results revealed that the photocatalytic degradation of bentazon in the presence of catalysts leads to a decrease in the absorption band as a function of the irradiation time. The photocatalytic degradation efficiency of bentazon with different catalysts follows the trend ZS > ZnO > Sm_2_O_3_. The nanocomposite shows superior photocatalytic activity as compared to the individual nanoparticles. This behavior can be ascribed to the rapid transfer of charge carriers, low recombination of photogenerated electron hole pairs and lower energy bandgap of nanocomposite [40]. The absorption spectra of bentazon in the presence of ZS nanocomposite is shown in Figure 5a. The photocatalytic degradation efficiency of ZnO, Sm_2_O_3_ NPs and ZS nanocomposites was 70%, 41% and 90%, respectively, after 140 min under UV light, as shown in Figure 5b.

Additionally, the kinetic study of photodegradation was examined using a first-order model as follows [36]:

(3) $$C_{t}=C_{o}e^{-kt}$$

(4) ln⁡(CoCt)=kt

where *k*, *C_o_* and *C_t_* represent the rate constant and the concentration of bentazon before illumination and after degradation under UV light as a function of time, respectively. A graph of *ln(C_o_/C_t_)* as a function of time (*t*) was plotted as shown in Figure 5c. The values of the rate constant *k* for all the prepared samples are displayed in Figure 5d. The obtained results indicate that the percentage degradation efficiency and rate constant *k* is higher for ZS nanocomposite as compared to individual NPs, so the ZS nanocomposite was selected for further investigating photocatalytic operational conditions.

#### 2.4.1. Effect of Photocatalyst Loading

**Figure 6:**
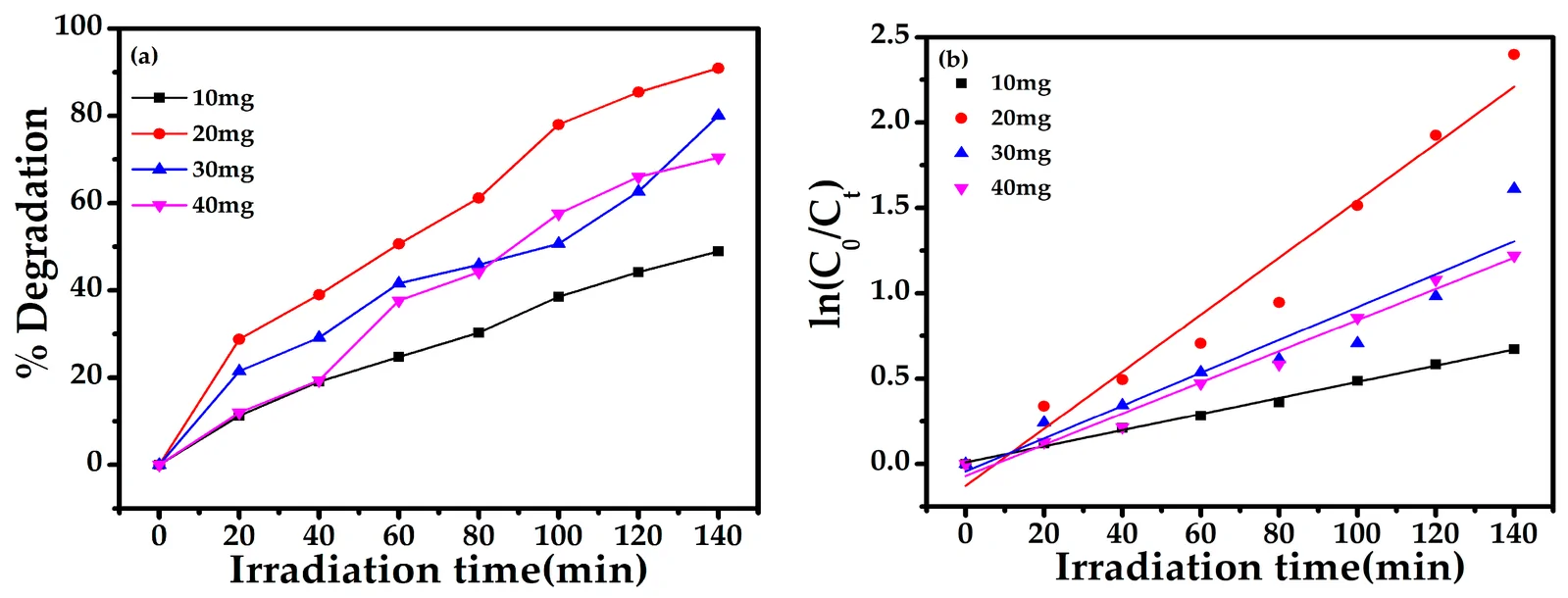
(**a**) Percentage degradation of bentazon at different amount of ZS nanocomposite and (**b**) kinetic plots of rate constant at different amounts of ZS nanocomposite.

The catalyst dosage in the photocatalytic process is an important factor that strongly affects the degradation of herbicides. In the photocatalytic experiment, the effect of the dosage of the catalyst on the photodegradation of bentazon herbicide was examined by changing the ZS nanocomposite amount (10–40 mg). After 140 min of UV irradiation, the degradation efficiency with a ZS catalyst loading of 10, 20, 30 and 40 mg under analogous conditions was 48%, 90%, 80% and 70%, respectively, as reported in Figure 6a. A graph of *ln(Co/Ct)* vs. time (*t*) was plotted at different catalyst loadings, as shown in Figure 6b. The values of rate constants (*k*) and R^2^ for the degradation of bentazon herbicide at different catalyst loadings are listed in Table 2. The results indicate that the degradation efficiency increased with the increase in catalyst loading of up to 20 mg, while the rate of the photo-destruction of herbicide was not improved when the catalyst amount increased more than 20 mg. The increment in photodegradation efficiency with the increase of catalyst amount is due to the increased availability of active sites, the higher photo capturing ability and the surface area of the catalyst. On the other hand, for a catalyst amount above 20 mg, due to the higher amount of the catalyst, the solution becomes cloudy and causes agglomeration, which reduces the penetration of UV light in the solution, leading to a reduction in the degradation efficiency.

#### 2.4.2. Effect of pH

**Figure 7:**
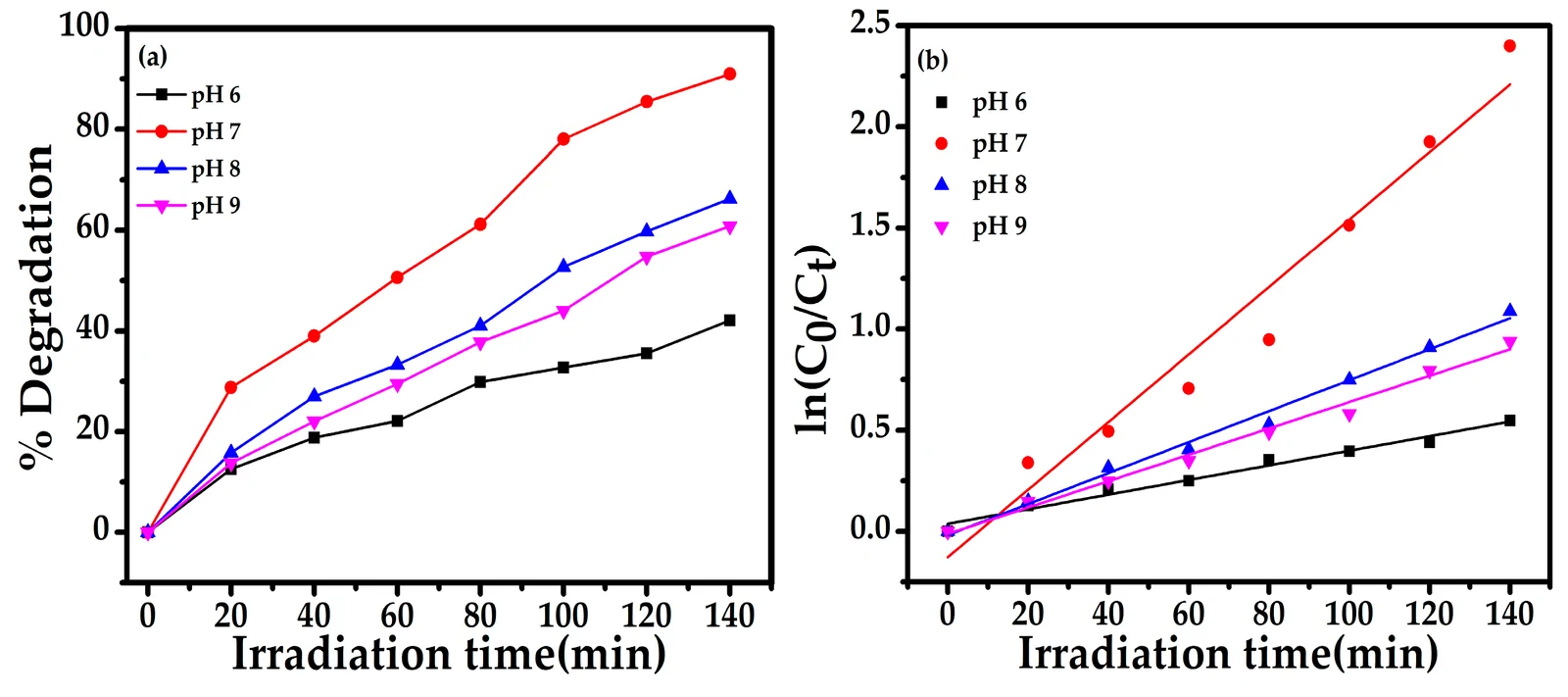
(**a**) Percentage degradation of bentazon at different pH of solution and (**b**) kinetic plots of rate constant at different pH of solution.

The pH variation of the solution is an essential factor for the degradation of herbicides. The degradation process of bentazon is due to several reaction mechanisms such as hydroxyl radical attack, oxidation through holes and reduction through electrons. These reaction mechanisms are linked with the pH of the solution and occur on the surface of the ZS nanocomposite. As the pH of the solution varies, it modifies the surface electrical charge of the catalyst, which affects the e^−^/h^+^ separation phenomena [41]. The effect of pH on photodegradation of bentazon was investigated by varying the pH of the solution (from 6 to 9) by using a solution of NaOH and HCl (1 M) at a fixed catalyst loading of 20 mg. Figure 7a shows the photodegradation of bentazon in the presence of ZS nanocomposite at different pH levels for 140 min of irradiation under UV light. From the experimental results, the degradation of bentazon was 42%, 90%, 66% and 60% at pH 6, 7, 8 and 9, respectively.

A graph of *ln(Co/Ct)* vs. time (*t*) was plotted at different pH values, as reported in Figure 7b. The values of rate constants (*k*) and R^2^ for the degradation of bentazon herbicide at different pH levels are listed in Table 2. By measuring the ζ-potential, and according to the Ohshima relationship [42], it is possible to determine the charge on the surface of nanoparticles. The ZS nanocomposite showed a ζ-potential equal to −45 mV and (by Ohshima relationship) the surface of the ZS particles is positively charged. At neutral pH, the production of reactive oxygen species (ROS) occurs and the bentazon is highly degraded. Decreasing the pH value to 6, more H^+^ ions are available to react with ROS ( $O_{2}^{•-}$ ) and hydrogen peroxide (H_2_O_2_) and gaseous oxygen (O_2_) are produced. Consequently, the degradation efficiency is decreased; in fact, the total ROS species are less available for photodegradation. On the contrary, increasing the pH to 8/9, more negative ions (OH^−^) are in the solution. These species could be attracted by the positively charged surface of the composite; by occupying the active sites responsible for ROS production, as a consequence, the degradation efficiency decreases.

#### 2.4.3. Effect of Bentazon Herbicide Concentration

**Figure 8:**
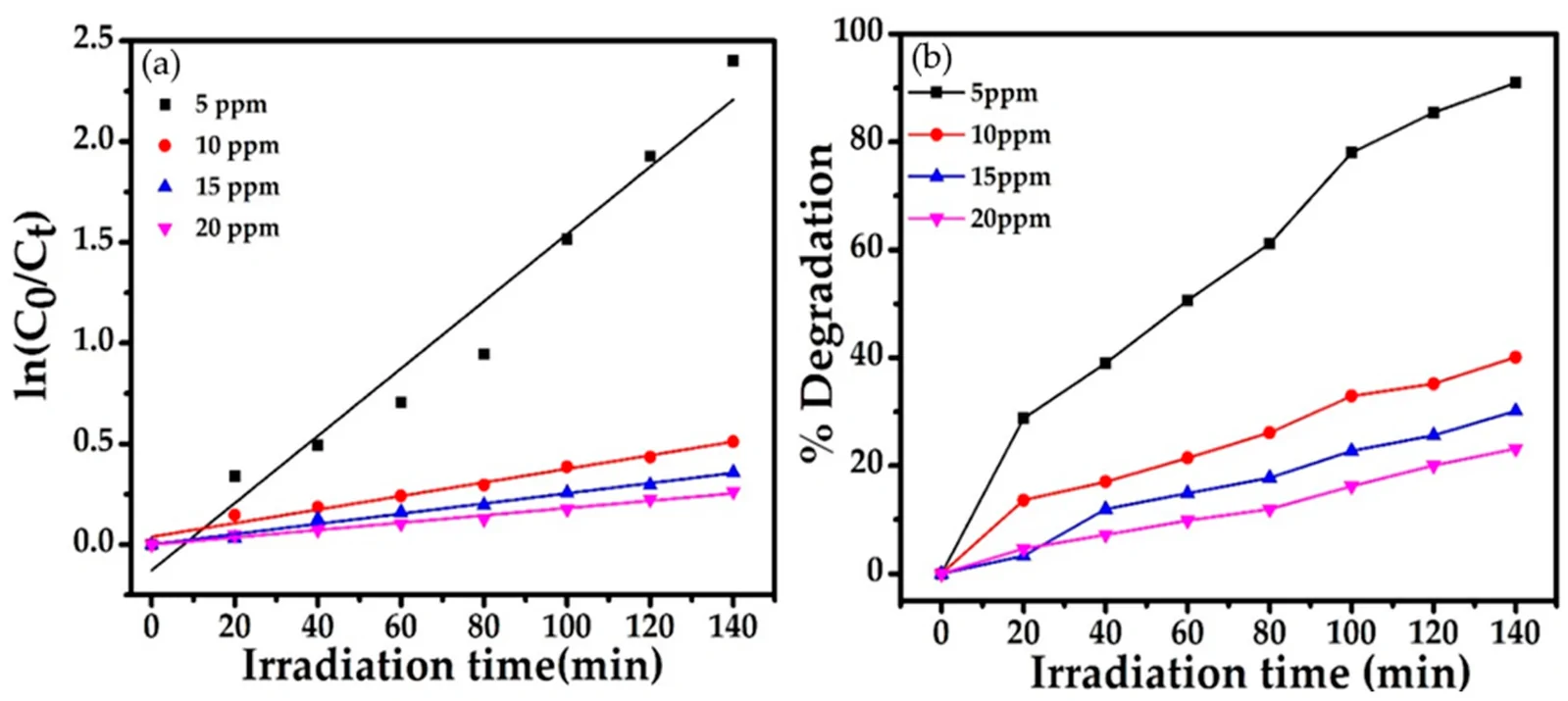
(**a**). Percentage degradation of bentazon at different concentrations of bentazon and (**b**) kinetic plots of rate constant at different concentrations of bentazon.

The photocatalytic degradation of bentazon herbicide was also investigated by varying the bentazon concentration in the range of 5–20 ppm with 20 mg of catalyst loading and a pH equal to 7. The photodegradation performance of ZS nanocomposite decreases with the increase in the bentazon concentration. The photocatalytic degradation of bentazon was 90%, 40%, 30% and 21% for a bentazon concentration of 5, 10, 15 and 20 ppm, respectively, as shown in Figure 8a. The graph of *ln(Co/Ct)* as a function of time (*t*) was plotted at different bentazon concentrations, as reported in Figure 8b. The values of the rate constants (*k*) and R^2^ for the degradation of bentazon herbicide at different bentazon concentrations are listed in Table 2. The photodegradation efficiency of ZS is decreased as the concentration of the bentazon is increased; this behavior might be due to the fact that as the concentration of the bentazon increases, a greater number of bentazon molecules absorb onto the photocatalyst surface, which reduces the light penetration, and there is less production of oxygen reactive species. According to the obtained results, a catalyst loading of 20 mg, pH value of 7, and bentazon concentration of 5 ppm are the optimal conditions for the maximum degradation efficiency of ZS.

#### 2.4.4. Scavenger Study

**Figure 9:**
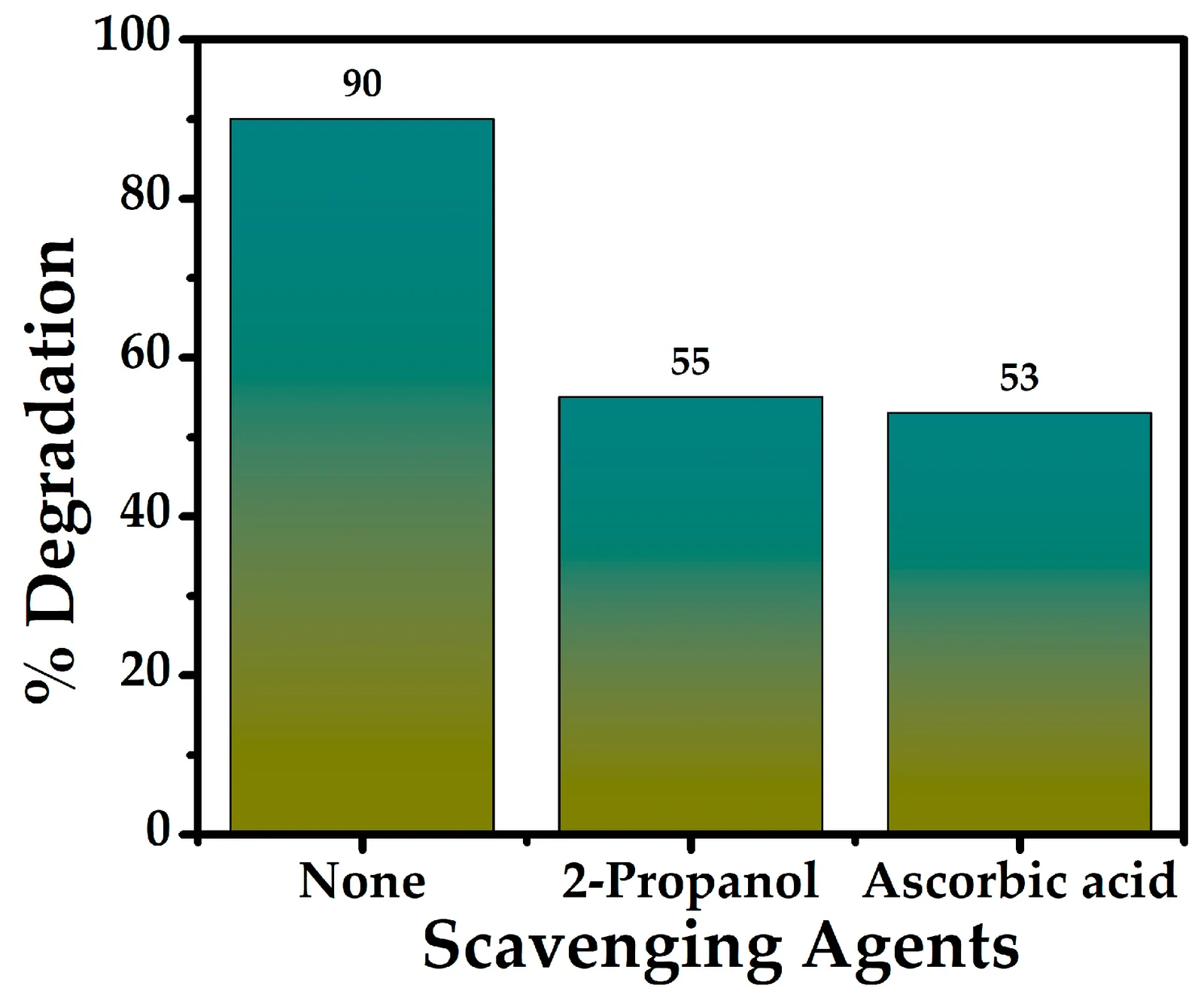
Effect of different scavengers on the degradation of bentazon.

A scavenger study was carried out to further verify the production of superoxide anions ( $O_{2}^{•-})\;$ and hydroxyl radicals (^•^OH) during photocatalytic activity and their role in degradation experiment. The degradation experiments were conducted by using 2-propanol and ascorbic acid as a scavenger for hydroxyl radicals (^•^OH) and superoxide radicals ( $O_{2}^{•-})$ . The scavenger experiments were conducted under optimization conditions (catalyst loading, 20 mg; pH of solution, 7; and bentazon concentration, 5 ppm) by adding scavengers to a bentazon–catalyst solution. The degradation experiment was conducted repeatedly for each scavenger and the obtained results are displayed in Figure 9. Without using any scavenger, the degradation efficiency of ZS nanocomposite was 90% for the removal of bentazon. However, in the presence of 2-propanol and ascorbic acid scavengers, the degradation efficiency reduces from 90% to 55% and 53%, respectively. The obtained experimental results indicate the reduction in the degradation of bentazon, which confirmed that the reactive species (^•^OH, $O_{2}^{•-}$ ) are produced during the experiment and play a major role in the photodegradation of bentazon.

#### 2.4.5. Photodegradation Mechanism

**Figure 10:**
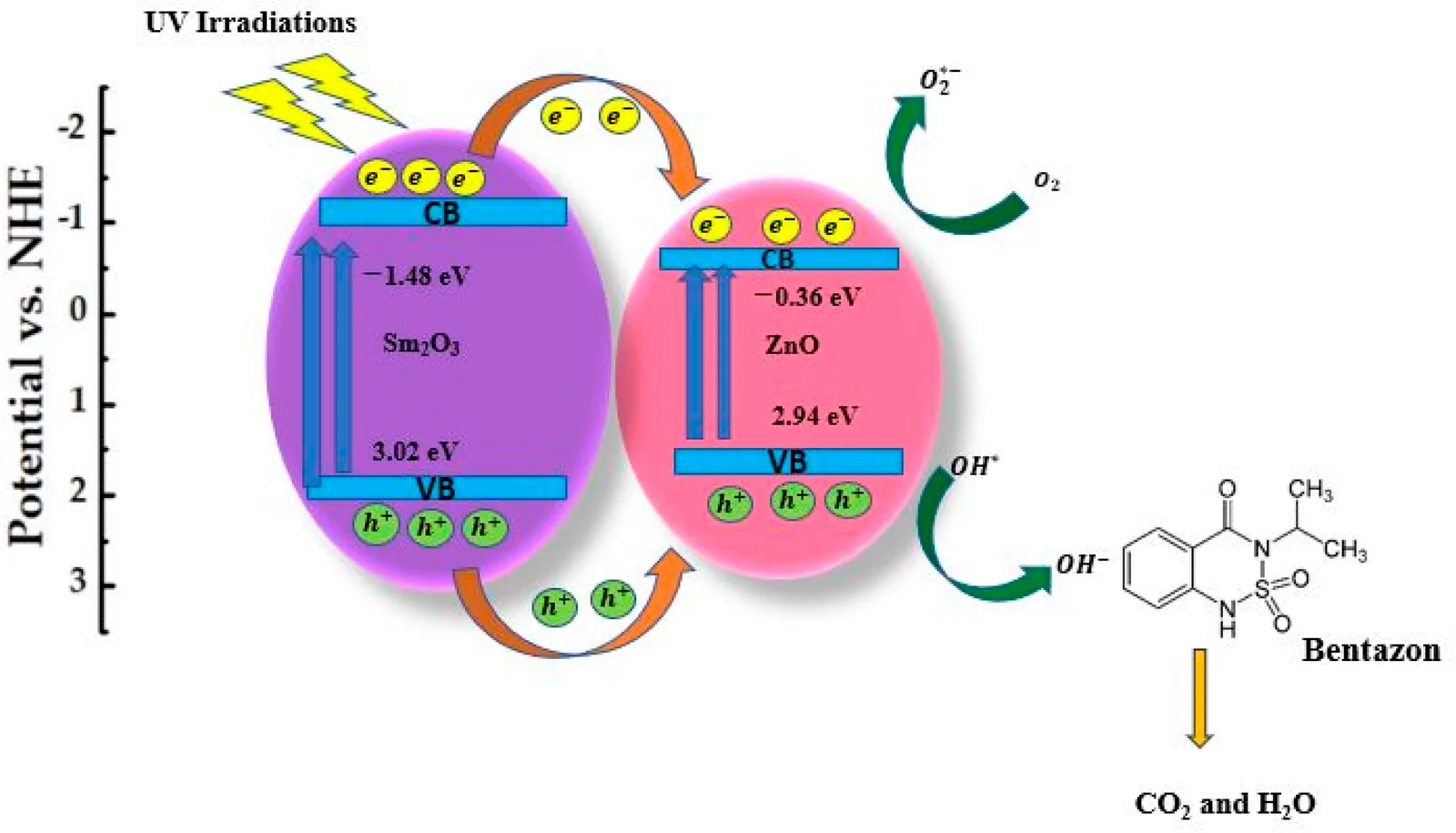
Schematic representation of photocatalytic mechanism of synthesized ZS nanocomposite against bentazon.

Generally, the photocatalysis process occurs through the following steps: photoexcitation, oxygen ion absorption, ionization of water and protonation of superoxide. When light falls on the catalyst, the electron hole pairs are generated. To understand the mechanism of flow of charge carriers, a schematic diagram is designed using band edge potentials (*E_VB_* and *E_CB_*) of ZnO and Sm_2_O_3_ as shown in Figure 10. The band edge potentials (*E_VB_* and *E_CB_*) of ZnO and Sm_2_O_3_ were computed from the equation given below [43]:

(5) $$E_{CB}=X-E_{H}-0.5E_{g}$$

(6) $$E_{VB}=Ecb+E_{g}$$

where *X* and *E_H_* represent the electronegativity and standard hydrogen potential. The *X* values for ZnO and Sm_2_O_3_ are 5.79 and 5.39 eV, respectively. The bandgap energies (*E_g_*) of ZnO and Sm_2_O_3_ are 3.30 eV and 4.54 eV, respectively, as calculated by UV-Vis data. The calculated values of *E_VB_* and *E_CB_* are 2.94 eV and −0.36 eV for ZnO and 3.02 eV and −1.48 eV for Sm_2_O_3_, respectively.

When light falls on the ZS photocatalyst, the transportation from valence band to conduction band occurs and electron and hole pairs are generated at the catalyst surface. Figure 10 represents the transportation mechanism of charge carriers for bentazon degradation. The electrons migrate from the highly negative conduction band of Sm_2_O_3_ to the lower negative conduction band of ZnO. The electrons and holes are separated effectively. At the same time, photogenerated holes from valence band of Sm_2_O_3_, due to higher band edge potential, can be transferred to the lower band edge of ZnO. The photogenerated electrons present in the conduction band react with oxygen molecules to form superoxide anions ( $O_{2}^{•-}$ ) while the holes present in the valance band react with hydroxyl ions to form hydroxyl radicals (^•^OH) [13,27]. The formed superoxide anions ( $O_{2}^{•-}$ ) and hydroxyl radicals (^•^OH) attach on bonds of the absorbed bentazon molecules to form byproducts such as CO_2_ and H_2_O [44]. The reactions mechanism is given below:

$$Sm_{2}O_{3}+h\nu ⟶e^{-}\left(Sm_{2}O_{3}\right)+h^{+}(Sm_{2}O_{3})$$

$$ZnO+h\nu ⟶e^{-}\left(ZnO\right)+h^{+}(ZnO)$$

$$e^{-}\left(ZnO\right)+O_{2}⟶O_{2}^{•-}$$

$$e^{-}\left(ZnO\right)+{2O}_{2}+2H^{+}⟶H_{2}O_{2}+e^{-}⟶OH^{*}$$

$$h^{+}\left(ZnO\right)+H_{2}O⟶OH^{*}$$

$$h^{+}\left(ZnO\right)+OH^{-}⟶ZnO+OH^{*}$$

$$O_{2}^{•-}+bentazon⟶Intermediate\;products$$

$$OH^{*}+bentazon⟶Intermediate\;products$$

The higher degradation efficiency of ZS nanocomposite is because of a low optical bandgap. A decrease in the bandgap of nanocomposite increases the ability of charge carriers production, exploiting lower light energy. Subsequently, photo-induced charge carriers increase the oxidation and reduction process in the conduction band and valence band, enhancing the photocatalytic activity.

A comparison of the photocatalytic activities of different individual oxides and nanocomposites for the degradation of bentazon reported in the literature is reported in Table 3.

## 3. Materials and Methods

### 3.1. Chemicals

Zinc nitrate hexahydrate [Zn(NO_3_)_2_·6H_2_O, purity 98%, crystal] and samarium nitrate hexahydrate [Sm(NO_3_)_3_·6H_2_O, purity 99%, crystalline solid in powder form] were used as precursors for the synthesis of ZnO, Sm_2_O_3_ nanoparticles and ZnO-Sm_2_O_3_ nanocomposites. Sodium hydroxide (NaOH, purity 97%, pallets) was employed as a precipitating agent. For the photocatalytic activity of prepared samples, bentazon (C_10_H_12_N_2_O_3_S, purity ≥ 98%, powder) was used as model pollutant in deionized water. Sodium hydroxide (NaOH) and hydrochloric acid (HCl, concentration 37%, density 1.2 g/mL) were used to obtain the required pH values. 2-propanol ((CH_3_)_2_CHOH), ≥99%) and ascorbic acid (C_6_H_8_O_6_, 99%) were used as scavengers. All these materials were purchased from Merck (Darmstadt, Germany) and used as received from Merck without any further refinement procedures.

### 3.2. Synthesis of ZnO, Sm_2_O_3_ Nanoparticles and ZnO-Sm_2_O_3_ Nanocomposites

**Figure 11:**
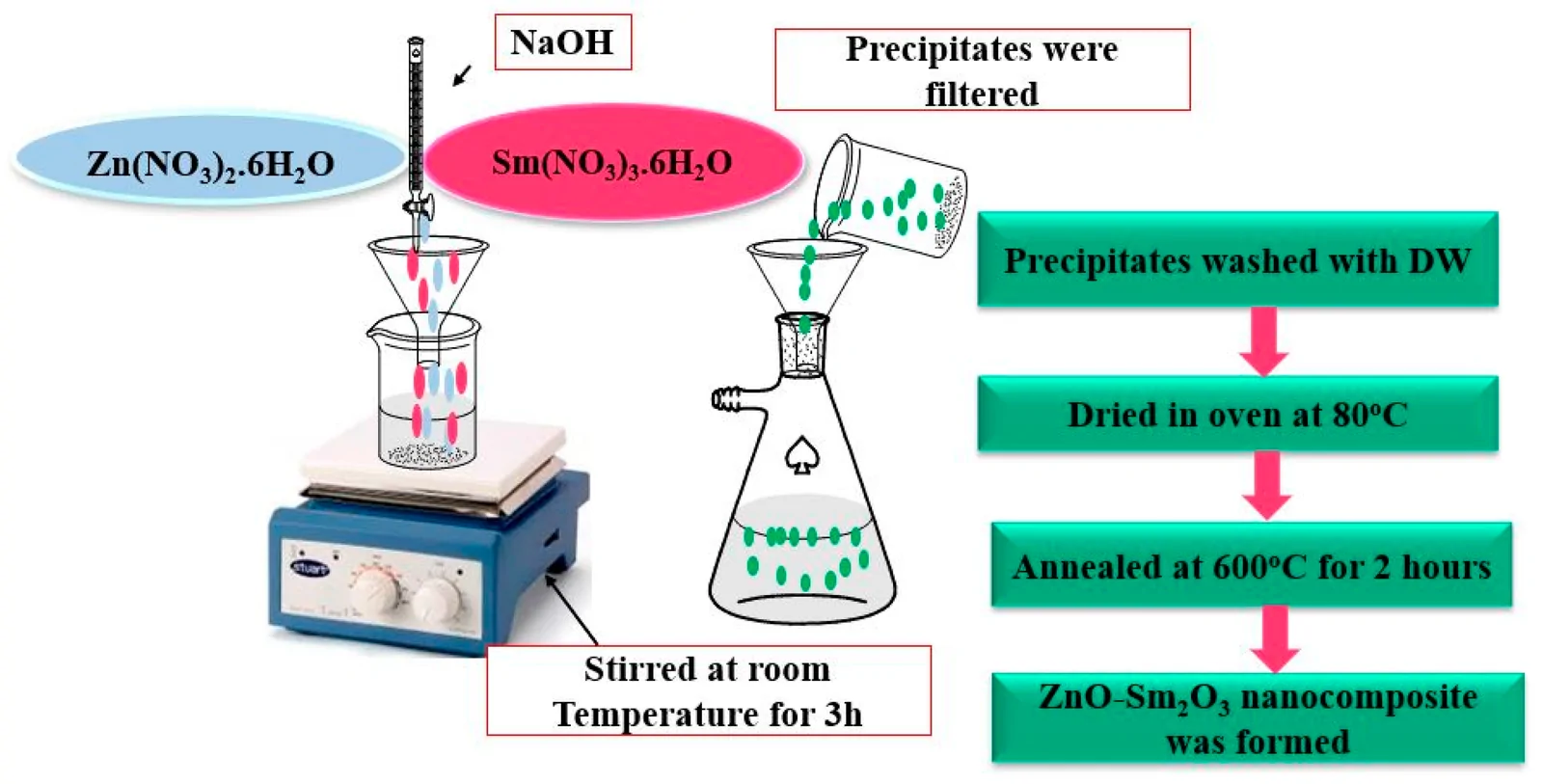
Schematic diagram of the synthesis of ZnO-Sm_2_O_3_ nanocomposite.

To synthesize ZnO-Sm_2_O_3_ binary nanocomposite, zinc and samarium salt of 0.1 M were mixed in molar ratio 1:1 in 100 mL distilled water (DW). For 100 mL solution, 1.48 g of zinc salt [Zn(NO_3_)_2_·6H_2_O] was mixed into 50 mL distilled water in one beaker and 2.22 g of samarium salt [Sm(NO_3_)_3_·6H_2_O] was mixed in another beaker. The two solutions were stirred separately for 1 h, then combined and magnetically stirred for 3 h. A solution of NaOH (1 M) was added dropwise in the suspension under continuous magnetic stirring at room temperature to achieve a pH of 9. After 3 h continuous stirring, the desired precipitates were formed. The obtained precipitates were washed twice with distilled water (DW), filtered and dried for 6 h at 80 °C in an oven. Lastly, the final products were put in a crucible and calcined at 600 °C in muffle furnace for 2 h and ground to make a fine powder. To prepare pure oxides nanoparticles of ZnO and Sm_2_O_3_, 1.48 g of zinc salt and 2.22 g of samarium salt were dissolved into 50 mL in two separate beakers and further proceed by using the same experimental conditions for comparative study. Figure 11 represents the schematic diagram of the experimental procedure.

### 3.3. Photocatalytic Degradation Experiment

The photocatalytic degradation efficiency of the grown ZnO, Sm_2_O_3_ NPs and ZS nanocomposite were investigated by using bentazon herbicide as a pollutant. In photocatalytic experiment, 20 mg catalyst powder was added to 60 mL solution of bentazon herbicide (5 ppm) at pH = 7. The solution was magnetically stirred for 1 h under dark to reach absorption/desorption equilibrium. The mixture solution was then irradiated using a mercury lamp (300 W) (Oriel Instruments, Newport, RI, USA). The light intensity of irradiation was about 126.5 mW/cm^2^, which was measured by a power meter (Thorlabs, model PM100D, Newton, NJ, USA) at wavelength 334 nm. The distance between the mixture solution and UV lamp was 20 cm. The concentration variation of bentazon herbicide was observed by using a double beam UV-Vis spectrophotometer (Lambda 750, Perkin-Elmer, Waltham, MA, USA) with a time interval of 20 min. The percentage degradation efficiency all samples was calculated by using the formula as given below [47]:

(7) $$Degradation\;efficiency=\left(C_{0}-C_{t}\right)/C_{0}\times 100$$

where *C_0_* and *C_t_* are the concentration of bentazon in the dark and after different times under UV light irradiation, respectively. To determine the optimization conditions, ZS nanocomposite was used for photodegradation experiments against bentazon at different catalyst loadings (10, 20, 30 and 40 mg), different pH values (6, 7, 8 and 9) and different concentrations of bentazon (5, 10, 15 and 20 ppm). The best results were obtained for the following conditions: catalyst loading of 20 mg, pH of 7 and bentazon concentration of 5 ppm. To confirm the role of reactive oxygen species, photocatalytic experiments were carried out by using optimization conditions by using 2-Propanol and Ascorbic acid as a scavenger.

### 3.4. Instrumentation

X-ray diffraction patterns were collected by means of a Philips X-Pert Pro 500 (Amsterdam, The Netherlands) diffractometer, with Cu Kα radiation (*λ* = 1.5418 Å) in the 20–80° 2θ range, with 1 s counting time and 0.02° step size. The optical and photocatalytic measurements were measured using a double beam UV-Vis spectrophotometer (Perkin Elmer UV/VIS/NIR spectrometer Lambda 750, Shelton, CT, USA). The photoluminescence spectra (PL) were detected by a photodetector coupled with a lock-in amplifier, as excitation source a LED with an emission wavelength of 430 nm (power 60 µW) was used. For surface morphology, samples were imaged using a TESCAN MIRA FE-SEM. The images were acquired using a secondary electron (SE) detector or, alternatively, an in-beam SE detector, setting the acceleration voltage at 5 kV and the probe current at 100 pA. The analysis was performed in the low vacuum pressurized chamber (10 Pa), while a working distance of around 8 mm was used. For ζ-potential measurements, a Zetasizer Ultra Red Label (Malvern Panalytical, Malvern, Worcestershire, UK) has been employed.

## 4. Conclusions

In summary, ZnO, Sm_2_O_3_ nanoparticles and ZnO-Sm_2_O_3_ nanocomposite were synthesized by the co-precipitation method and were used as catalysts for the photodegradation of bentazon herbicide. Compared with the individual oxides, the bentazon degradation ability of ZS nanocomposite was much higher under UV light irradiations. The formation of a heterojunction of ZnO and Sm_2_O_3_ leads to a synergistic effect that helps the charge transportation and inhibits the recombination of photoinduced charge carries that increase the photocatalytic performance of ZS nanocomposite. The degradation efficiency of ZS nanocomposite was 90% at acquired optimization conditions for the bentazon decomposition in 140 min under UV light irradiations. The role of reactive species during the photocatalytic experiment was accessed by using different scavengers, which reveals that reactive species play an active role in the degradation of bentazon herbicide. Our findings suggest that ZS nanocomposite is a promising applicant for the removal of organic pollutants from wastewater and improving water quality. The coprecipitation method is a simple and highly scalable industry synthesis process, consisting in mixing the precursors salts and changing the pH by NaOH. The cost of the materials and the energy necessary to produce a quantity of composite suitable for one liter of polluted water is about EUR 0.90, which is a competitive cost for real-world wastewater treatment applications. In conclusion, the present study reports the feasibility for the eco-friendly and low-cost synthesis of ZS nanocomposite, with high adsorption capacity and excellent photocatalytic activity, by developing the heterojunction photocatalyst system. Hence, the ZS nanocomposite can be used for practical implementation in industries for the treatment of wastewater.

## Figures and Tables

**Table 1:** Microstructural parameters of ZnO, Sm_2_O_3_ NPs and ZS nanocomposite.

Samples	a (Å) = b (Å)	c ( A° )	c/a	Volume ( ${Å}^{3}$ )	d-Spacing	Average Crystallite Size (nm)	Dislocation Density 10^−3^ (nm^−2^)
Nanoparticles							
ZnO Sm_2_O_3_	3.244 10.897	5.197 -	1.601 1	47.368 1294.031	1.829 2.071	21.767 45.361	2.210 0.485
ZS nanocomposite							
ZnO Sm_2_O_3_	3.246 10.916	5.198 -	1.601 1	47.441 1299.167	1.924 2.281	33.838 45.866	1.5944 0.62301

**Table 2:** Photodegradation kinetic parameters at different experimental conditions for ZS nanocomposite against bentazon herbicide.

Catalyst Loading	Bentazon Concentration	pH	% Degradation	K (min^−1^)	R^2^	y = a + bx
10 mg	5 ppm	7	49	0.00472	0.9958	0.0094 + 0.0047x
20 mg	--	--	90	0.01669	0.9572	−0.1273 + 0.0166x
30 mg	--	--	80	0.00961	0.8790	−0.0429 + 0.0096x
40 mg	--	--	70	0.00912	0.9829	−0.0693 + 0.0091x
20 mg	5 ppm	6	42	0.00361	0.9779	0.0379 + 0.0036x
--	--	7	90	0.01669	0.9585	−0.1273 + 0.0166x
--	--	8	66	0.00765	0.9704	−0.0174 + 0.0076x
--	--	9	60	0.00651	0.9875	−0.0117 + 0.0065x
20 mg	5 ppm	7	90	0.01669	0.9585	−0.1273 + 0.0166x
--	10 ppm	--	40	0.00341	0.9780	0.0392 + 0.0033x
--	15 ppm	--	30	0.00253	0.9871	0.0017 + 0.0025x
--	20 ppm	--	21	0.00182	0.9872	−0.5076 + 0.0018x

**Table 3:** The comparison study of photodegradation efficiency of different metal oxide nanocomposite materials against bentazon herbicide.

Photocatalyst	Source	Irradiation Time (min)	Degradation Efficiency (%)	Refs.
ZnO	UV light	60	32%	[45]
CuO	-	60	10%	-
ZnO	-	140	70%	Present work
Sm_2_O_3_	-	140	40%	Present work
N–TiO_2_–PMAA-g-PVDF/PAN	-	180	90%	[46]
Fe_2_O_3_-TiO_2_	-	120	51%	[1]
NiO-ZnO	-	100	70%	[7]
ZnO-Sm_2_O**_3_**	-	140	90%	Present work

## Data Availability

All the data are published with this manuscript.
