# Correction: Yasmeen et al. Study of Photodegradation of Bentazon Herbicide by Using ZnO-Sm_2_O_3_ Nanocomposite Under UV Light. *Int. J. Mol. Sci.* 2024, *25*, 13319

**DOI:** 10.3390/ijms27177599

**Published:** 2026-08-25

**Authors:** Sadaf Yasmeen, Luca Burratti, Leonardo Duranti, Antonio Agresti, Paolo Prosposito

**Affiliations:** 1 Industrial Engineering Department, University of Rome Tor Vergata, Via del Politecnico 1, 00133 Rome, Italy; sadaf.yasmeen@students.uniroma2.eu (S.Y.); paolo.prosposito@uniroma2.it (P.P.); 2 Department of Engineering and Sciences, Mercatorum University, Piazza Mattei 10, 00186 Rome, Italy; 3 Department of Chemical Science and Technologies, University of Rome Tor Vergata, Via Della Ricerca Scientifica 1, 00133 Rome, Italy; leonardo.duranti@uniroma2.it; 4 Department of Electronic Engineering, University of Rome Tor Vergata, Via del Politecnico 1, 00133 Rome, Italy; antonio.agresti@uniroma2.it

In the original publication [1], there was a mistake in Figure 2 caption. Figure 2a has appeared in the author’s previous publication and needs to be cited here. The correct Figure 2 caption is as follows. The authors state that the scientific conclusions are unaffected. This correction was approved by the Academic Editor. The original publication has also been updated.

**Figure 2.** UV-Vis absorption spectra of (**a**) ZnO (reprinted from Figure 5b in previous article [7]), (**b**) Sm_2_O_3_ nanoparticles and (**c**) ZS nanocomposite; insets show Tauc’s plot of prepared samples for energy bandgap estimation.
